# Three-dimensional positioning and control of colloidal objects utilizing engineered liquid crystalline defect networks

**DOI:** 10.1038/ncomms8180

**Published:** 2015-05-21

**Authors:** H. Yoshida, K. Asakura, J. Fukuda, M. Ozaki

**Affiliations:** 1 Division of Electrical, Electronic and Information Engineering, Osaka University, 2-1 Yamada-oka, Suita, Osaka 565-0871, Japan; 2 Nanosystem Research Institute, National Institute of Advanced Industrial Science and Technology (AIST), 1-1-1 Umezono, Tsukuba 305-8568, Japan

## Abstract

Topological defects in liquid crystals not only affect the optical and rheological properties of the host, but can also act as scaffolds in which to trap nano or micro-sized colloidal objects. The creation of complex defect shapes, however, often involves confining the liquid crystals in curved geometries or adds complex-shaped colloidal objects, which are unsuitable for device applications. Using topologically patterned substrates, here we demonstrate the controlled generation of three-dimensional defect lines with non-trivial shapes and even chirality, in a flat slab of nematic liquid crystal. By using the defect lines as templates and the electric response of the liquid crystals, colloidal superstructures are constructed, which can be reversibly reconfigured at a voltage as low as 1.3 V. Three-dimensional engineering of the defect shapes in liquid crystals is potentially useful in the fabrication of self-healing composites and in stabilizing artificial frustrated phases.

## Abstract (web summary)

Topological defects can be used not only to modify the properties of liquid crystals but also as scaffolds to build new structures by trapping particles. Here, Yoshida *et al*. construct three-dimensional colloidal superstructures in a nematic host, which are reconfigurable in an electric field.

## Graphical abstract

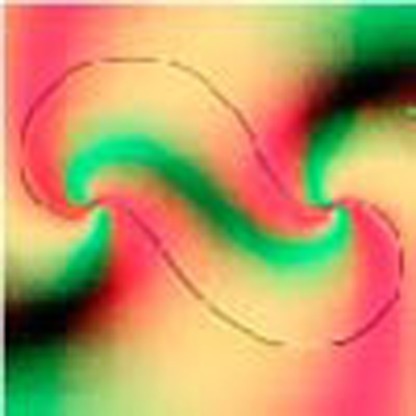

Liquid crystals find applications in diverse scientific disciplines, ranging from optics, electronics, mechanics, biology and cosmology^1^,^2^,^3^,^4^,^5^. Their usefulness derives from their broken rotational symmetry, which gives rise to spontaneous alignment that can be controlled through boundary conditions at interfaces or by an external field. It is well known that liquid crystal displays operate by controlling the birefringence in a slab of nematic liquid crystal, which is the simplest, non-trivial ordered phase of a liquid crystal. In a nematic liquid crystal, rod-like molecules are packed such that they have no positional order but have orientational order with the long axes of the molecules pointing along the ‘director', defined by a unit vector, **n**, with head–tail symmetry (that is, **n** and **–n** are equivalent).

The vectorial nature of the director (**n**=***−*****n**) enables nematic liquid crystals to accommodate various kinds of topological defects, or singularities in the director field, **n**(**r**), also known as disclinations^6^. The fact that liquid crystals typically show textures with characteristic length scales in the micrometre range have rendered them particularly attractive for the testing of various topological theorems. Nematic liquid crystals confined in spheres spontaneously form bulk and surface defects obeying predictions from the Gauss–Bonnet and Poincaré–Hopf theorems of topology^7^,^8^. In recent times, the same theorems were confirmed in an inverted system, in which colloidal particles having different topological genera were dispersed in liquid crystals^9^. Moreover, the disclinations were found to self-organize into knotted or linked structures, in response to the knot in the colloidal particle itself, or chirality of the surrounding liquid crystal host^10^,^11^.

Although disclinations had historically been subjects of study of purely scientific interest, some recent studies have shown interest in disclinations as tools to be exploited technologically. For example, optical vortices, which are light beams carrying a topological charge, are generated via light–matter interactions in a liquid crystal slab with topological defects^12^,^13^; colloidal particles doped in liquid crystals generate a network of disclinations that confer the composite the properties of a self-healing gel^14^,^15^; and disclinations have been used as scaffolds in which to trap conductive or plasmonic particles to potentially realize three-dimensional micro-wires or tunable metamaterials^16^,^17^,^18^,^19^,^20^. The potential of liquid-crystal-based composites originates from the fact that they can self-organize into structures that are not easily attainable via conventional top–down fabrication technology^21^. One of the challenges for their widespread use, however, is to develop a means of controlling the disclination numbers and shapes. Fabrication of complex-shaped structures are not only difficult and cumbersome, but the disclinations generated by such structures are bound to their surfaces, rendering positional control and tuning difficult^9^,^10^,^11^,^22^. On the other hand, although patterned-rubbing-based methods can generate disclinations running through the bulk in a flat liquid crystal slab^19^,^20^,^23^,^24^, studies reported to date have only succeeded in generating defect lines with simple (linear or circular) shapes and the possibility of tuning the disclination shapes in three dimensions has not been discussed.

In this work, we demonstrate controlled generation of a three-dimensional disclination network by confining a liquid crystal slab between two substrates that possess topological surface anchoring conditions, that is, the substrates contain a singular point or defect around which the orientational easy axis rotates by an integral multiple of *π*. Interestingly, the disclination line numbers are controlled by the topological charge or strength^6^ of the defect (the number of 2*π* rotations), whereas their shapes are controlled by the far-field director distribution surrounding the defect. This role sharing between defect generation and shape morphing is exploited to engineer disclinations into non-trivial shapes. Circular, spiral-like and chiral disclination networks are generated in an achiral liquid crystal, pentylcyanobiphenyl (5CB), and three-dimensional colloidal superstructures are constructed by using the disclination networks as templates. In addition, as the disclinations exist in the bulk of the liquid crystal slab, efficient electric-field tuning is demonstrated, requiring only 1.9 V to shrink the length by ∼12%. The concept of defect engineering we propose introduces the possibility of fabricating liquid crystal composites with controlled structures that may be used in a variety of applications.

## Results

### Disclination generation by topological surface anchoring

Projection exposure photoalignment is employed to fabricate the topologically patterned substrates. A linearly polarized light passing through a bow-tie-shaped slit is imaged onto a glass substrate coated with a photoaligning layer by a combination of a tube lens and an objective lens. The sample stage and polarizer are rotatable and are controlled electronically so that the ratio of their rotation speeds, *ω*_polarizer_/*ω*_stage_, is equal to an integer^13^; the strength of the defect, *s*, is then given by (1−*ω*_polarizer_/*ω*_stage_). The combination of *s* and the initial polarization direction, *c*, where *c*=0 is set parallel to the slit opening, defines the orientation pattern for a given slit shape. For the straight bow-tie-shaped slit used in our first experiment, the easy axis at a given position is given by *n*=(cos*ϕ*, sin*ϕ*, 0), where *ϕ*=*s* tan^−1^(*y*/*x*)+*c*, with the *x*–*y* plane taken to be parallel to the substrate with the origin at the defect core (Supplementary Fig. 1). A cell is constructed using two substrates containing the same pattern and the 5CB is inserted in the cell via capillary forces. The resulting textures are observed by means of polarized optical microscopy (POM) and two-photon excitation microscopy (TPEM)^25^.

**Figure 1:**
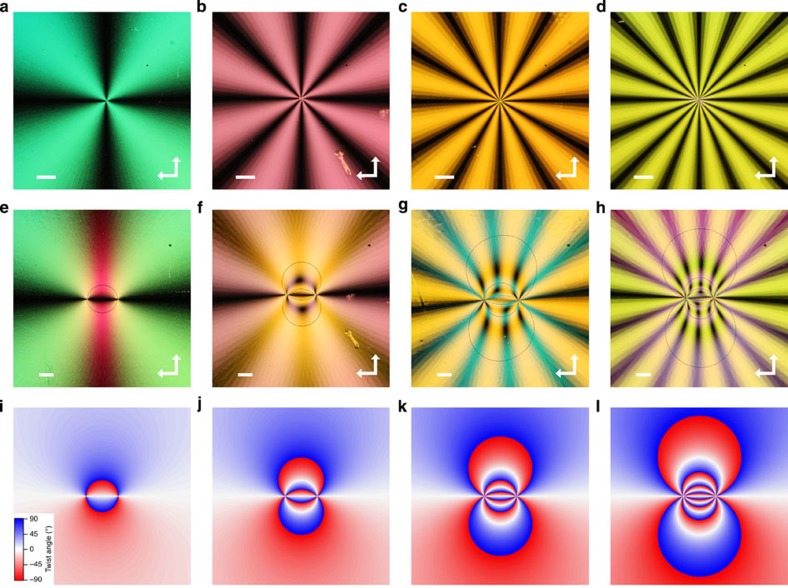
Disclination generation from topological patterns created with a linear slit. POM images of liquid crystal cells with orientational patterns (*s, c*)=(1, 0) (**a**), (*s, c*)=(2, 0) (**b**), (*s, c*)=(3, 0) (**c**) and (*s, c*)=(4, 0) (**d**). The lateral separation of the two substrates is 0. Arrows indicate the direction of polarizers. Scale bars, 100 μm. (**e**–**h**) POM images of the same sample as in **a**–**d** after separating the patterns on the two substrates by 200 μm. Spatial distribution of the twist angle for substrates with orientational patterns (*s, c*)=(1, 0) (**i**), (*s, c*)=(2, 0) (**j**), (*s, c*)=(3, 0) (**k**) and (*s, c*)=(4, 0) (**l**), calculated by placing the defect centres of the top and bottom substrates at (0.41X, 0.5X) and (0.59X, 0.5X), respectively, where X is the width of the figure.

Figure 1 shows POM images of the samples patterned with different defect strengths. When the patterns on the two substrates are aligned (Fig. 1a–d), black stripes appear between crossed polarizers where the director is parallel to one of the polarizers. The number of the dark stripes is equal to 4|*s*|^6^. As the patterns on the two substrates are laterally separated, disclination lines that appear as thin dark lines are seen connecting the two defect centres, with their number being equal to 2|*s*| (Fig. 1e–h). A movie recorded during this process shows that the disclinations appear as soon as the patterns are separated, and increases in size while retaining their shape as the separation increases (Supplementary Movie 1). The different colours observed in Fig. 1 corresponds to the liquid crystal being observed at different temperatures and thus possessing different birefringence. Because of the strong anchoring imposed, the patterns are stable even at temperatures close to the clearing point.

The number of disclinations emanating from an imprinted defect is governed by *s* through the conservation law of topological charge. When one considers a plane parallel to and in the vicinity of the cell substrate, one can safely define the strength of a disclination line penetrating it, because the substrate imposes planar alignment and, therefore, out-of-plane director distortions in the vicinity of the substrates are extremely energetically unfavourable. The strength of a disclination line is +1/2 or −1/2, as a disclination line of higher strength splits into lines of strength +1/2 or −1/2, reducing the Frank elastic energy associated with the director distortions^6^. In addition, only disclination lines of the same sign remain, as a pair of disclination lines of strength +1/2 and −1/2 mutually annihilate to reduce the elastic energy^6^. Therefore, the number of disclination lines emanating from an imprinted defect of strength *s* must be 2|*s*|, to conserve the topological charge. Because of technical difficulties in fabricating perfect defects of high strength^26^, one often encounters imperfect defects in which the defect core is split into defects of strength 1/2 (ref. 27). However, the overall topological charge, and hence disclination numbers, are also conserved in this case, as an imperfect defect of strength *s* splits into 2|s| defects of strength 1/2.

It is also interesting to note that this law applies when the substrates composing a cell are patterned with differing values of *s*. Supplementary Fig. 2 shows a sample fabricated by sandwiching two substrates patterned with *s*=−1 and −2 defects. As predicted, 2|*s*| disclinations emanate from each defect; however, as there is a mismatch in the defect strength, the number of disclinations connected between the two defect centres is limited to the smaller value of |*s*|. The unconnected disclinations extend outwards past the region covered by the orientational pattern, resulting in a network that is less symmetric.

The shapes of the disclination lines are determined from the far-field orientational pattern surrounding the defect core. Let Ψ_t_(*x*, *y*) and Ψ_b_(*x*, *y*) denote the azimuthal angles of the director at the top and bottom surfaces, respectively, at a given lateral position, (*x*, *y*). When Ψ_t_(*x*, *y*)≠Ψ_b_(*x*, *y*), the director is twisted along the cell normal and relaxes the Frank elastic energy^6^. If Ψ_t_(*x*, *y*)−Ψ_b_(*x*, *y*) exceeds *π*/2, the handedness of the twist reverses, so that Ψ_t_(*x*, *y*)−Ψ_b_(*x*, *y*) becomes –*π*/2 (note that the head and tail equivalence of the director^6^ allows this shift of Ψ_t_(*x*, *y*)−Ψ_b_(*x*, *y*) by *π*. As 5CB is achiral, the elastic energy of a twist distortion with Ψ_t_(*x*, *y*)−Ψ_b_(*x*, *y*)=*π*/2 is equal to that for Ψ_t_(*x*, *y*)−Ψ_b_(*x*, *y*)=–*π*/2). A discontinuous shift of the twist-handedness must be accompanied by a disclination line, around which the director rotates by *π*. Calculation of Ψ_t_(*x*, *y*)−Ψ_b_(*x*, *y*) for different imprinted defect strengths successfully reproduces the disclination line shapes and positions (Fig. 1i–l).

**Figure 2:**
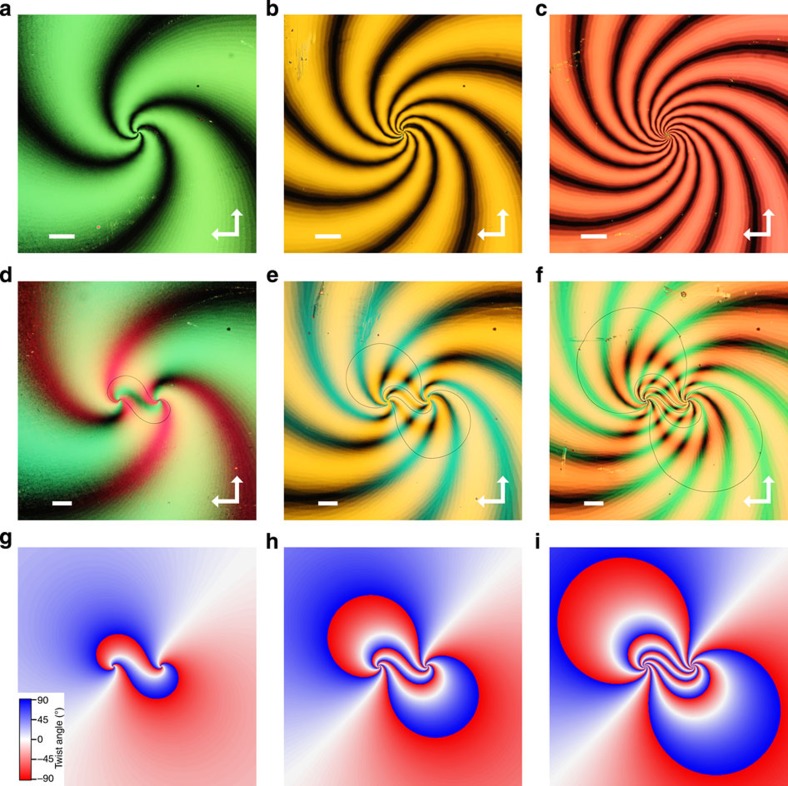
Disclination generation from topological patterns created with a spiral slit. POM images of liquid crystal cells with disclination strengths (*s, c*)=(1, 0) (**a**), (*s, c*)=(2, 0) (**b**) and (*s, c*)=(3, 0) (**c**), when the lateral separation of the two substrates is 0. Scale bars, 100 μm. (**d**–**f**) POM images of the same sample as in **a**–**c** after separating the patterns on the two substrates by 200 μm. Spatial distribution of the twist angle for substrates with spiral orientational patterns (*s, c*)=(1, 0) (**g**), (*s, c*)=(2, 0) (**h**) and (*s, c*)=(3, 0) (**i**), calculated by placing the defect centres of the top and bottom substrates at (0.4X, 0.5X) and (0.6X, 0.5X), respectively, where X is the width of the figure.

The role sharing of defect generation and shape control by the topological charge and far-field orientational pattern allow for disclination numbers and shapes to be engineered. We change the slit shape from a straight bow tie to a logarithmic spiral (Supplementary Fig. 3). The topological charge is still defined by the relative speeds of the two rotating stages, but the far-field director now follows the pattern defined by the slit, that is, *ϕ*=*s* {tan^−1^(*y*/*x*)±ln ((*x*^2^+*y*^2^)^1/2^)}+*c* (the sign of the second term in the logarithm varies depending on the direction from which the sample is observed; Fig. 2a–c). Figure 2d–f shows POM images of the disclination lines created from spiral patterns with *s*=1, 2 and 3 (*c*=0). Similar to Fig. 1, the number of disclinations varies according to the topological charge or strength of the imprinted defect pattern. However, the resultant shape is different from the cases in which a linear slit is used and is clearly affected by the far-field director profile. The obtained shapes are again in agreement with those predicted from the spatial distribution of the twist angle (Fig. 2g–i).

### Templated assembly of colloidal particles

**Figure 3:**
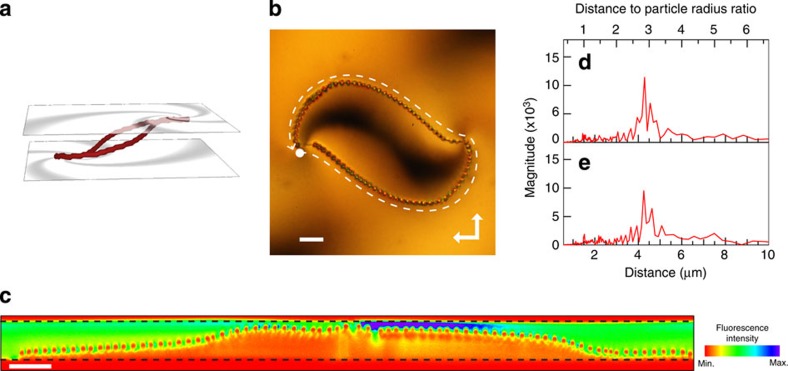
Templated three-dimensional assembly of colloidal particles. (**a**) Three-dimensional profile of the disclination network formed between spirally patterned substrates with (*s, c*)=(1, 0), calculated from a Landau–de Gennes theory. The cell gap has been extended twofold for clarity. (**b**) POM image of the disclination network decorated with colloidal particles of 3 μm in diameter. The white dashed line indicates the direction along which the cross-sectional profile was measured. Scale bar, 20 μm. (**c**) Cross-sectional TPEM profile along the disclination shown in **b**. The dashed lines mark the substrate boundaries. (**d**,**e**) FFT magnitude of the TPEM intensity along the left and right particle chains shown in **c**.

The twist distributions mentioned above can predict only two-dimensionally projected disclination shapes, although they are, in reality, three-dimensional, because of the cell asymmetry in the depth direction. Simulation of the order parameter tensor based on the Landau–de Gennes free energy confirms that the disclination lines do run three dimensionally within the cell, connecting the two defect centres imprinted on each substrate (Fig. 3a, see also Supplementary Fig. 4 for disclination networks generated using substrates with different boundary conditions). This allows three-dimensional composites to be constructed by using the network of disclination lines as a template. We decorate the disclination lines with silica microspheres of 3-μm in diameter and observe their distribution by means of POM and TPEM (a dichroic dye is also doped in the liquid crystal to detect the particle positions from the fluorescence contrast). The silica particles are coated with DMOAP (dimethyloctadecyl[3-(trimethoxysilyl)propyl] ammonium chloride) to impose vertical alignment at the particle surface^28^, and so the director distortion around the particle causes a twisted disclination ring to surround the particle. The disclination-decorated particles are then attracted towards the surface-induced disclination via elastic interactions, minimizing the director deformation^18^. Figure 3b shows the POM image of the colloidal superstructure constructed using substrates patterned with a spiral defect of strength 1. TPEM confirms that the particles are distributed three-dimensionally within the cell, with a roughly regular spacing between two adjacent particles (Fig. 3c, see also Supplementary Movie 2). As chain structures comprising a smaller number of particles were observed with similar inter-particle spacings, we infer that the spacing between the particles is the potential minimum at which the long-range attractive and short-range repulsive forces (due to director deformation) are balanced for particles located on a disclination line^29^. Fourier (Fig. 3d,e) and space-frequency (Supplementary Fig. 5) analyses of the particle chain yield 4.3 μm as the dominant inter-particle distance. This corresponds to an inter-particle distance to particle radius ratio of ∼2.9, which is slightly larger than 2.46, the value obtained for particle chains formed in a uniformly aligned nematic liquid crystal^29^. The structure at equilibrium is stable and is not destroyed unless the host nematic liquid crystal reaches the isotropic phase, because of the large trapping potential (estimated to be of the order of ∼500 *k*_B_*T* for the 3-μm particles dispersed in 5CB; ref. 19) imposed on the particles.

**Figure 4:**
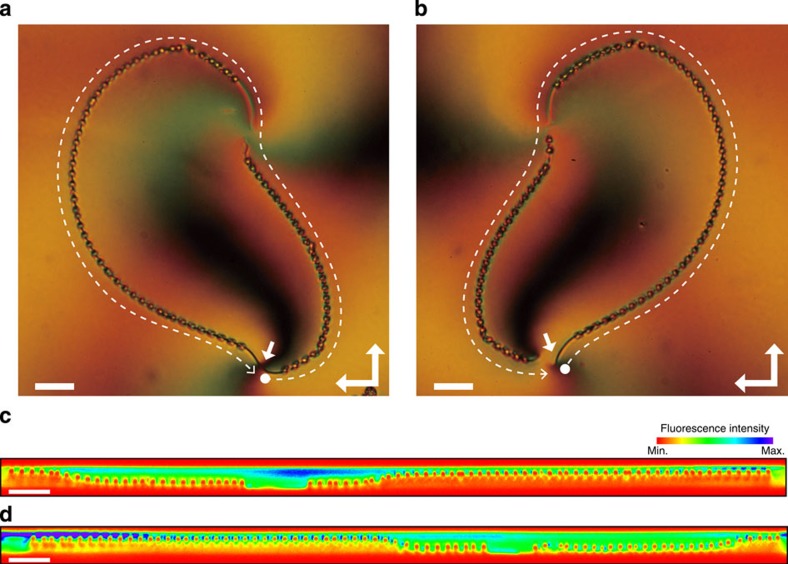
Chiral assembly of colloidal particles. (**a**,**b**) Colloidal particles trapped in the disclination lines generated from spiral defects of (*s, c*)=(1, *π*/6) and (1, 0) (**a**), and (*s, c*)=(1, −*π*/6) and (1, 0) (**b**), on the front and rear substrates, respectively. The white dashed lines indicate the direction along which the cross-sectional profile was measured and the solid white arrows indicate the defect centre that exists on the front substrate. Scale bars, 20 μm. (**c**,**d**) TPEM profile along the disclination line, corresponding to **a**,**b**. The top and bottom of each figure corresponds to the front and rear substrates, respectively.

The shape of the disclination network can be further tuned by adjusting the initial angles (*c*) on the top and bottom substrates. Offsetting the initial angles of the two substrates induces symmetry breaking, which results in the generation of asymmetric disclination lines connecting the defect centres. Figure 4 shows the disclination patterns fabricated by sandwiching spirally patterned substrates with (*s*, *c*)=(1, *π*/6) and (1, 0), and (*s*, *c*)=(1, −*π*/6) and (1, 0), on the front and rear substrates, respectively. One of the disclination lines appears shorter than the other and the TPEM profile along the disclinations shows that the vertical positions of the particles are inverted with respect to the path lengths. The two assemblies cannot be superposed regardless of how they are rotated or translated, implying they are chiral. This wide structural controllability is another consequence of the role sharing between the defect generation and shape morphing.

### Electric-field tuning of disclination shapes

**Figure 5:**
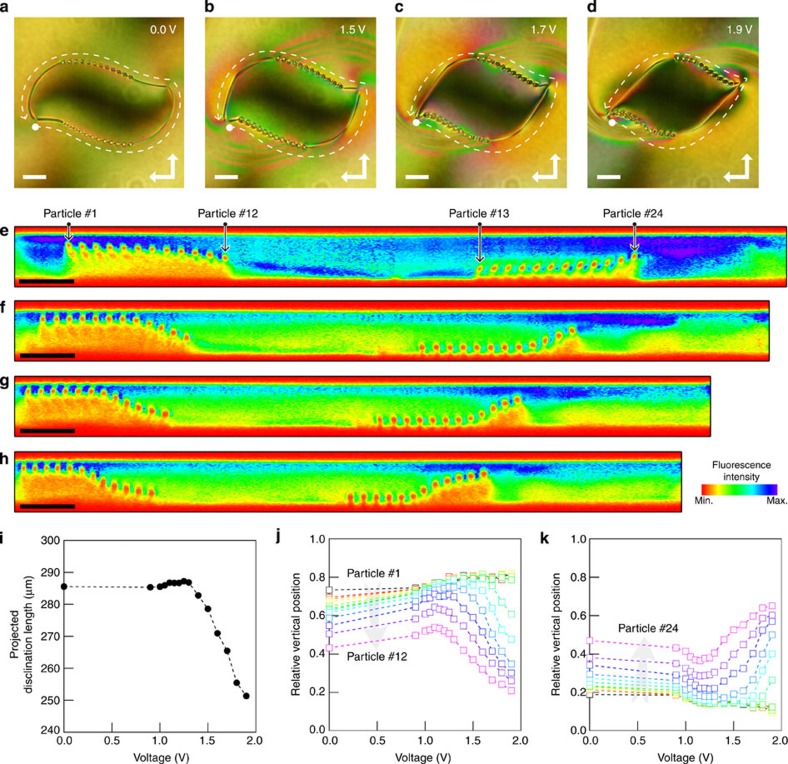
Field-induced reconfiguration of disclination shapes and particle positions. (**a**–**d**) POM images of the colloid-decorated disclination network constructed using two substrates with spiral defects of (*s, c*)=(1, 0) at various applied voltages. White dashed lines indicate the direction along which the cross-sectional profile was measured. Scale bars, 20 μm. (**e**–**h**) TPEM profiles along the disclination lines in **a**–**d**. (**i**) Voltage dependence of two-dimensionally projected disclination length measured from the POM images. Relative vertical positions of the particles in the lower (**j**) and upper (**k**) chains in the POM images, where the cell gap is normalized to 1. Particles are numbered from 1 to 24 in the order or appearance along the profile path (see **e**).

Finally, active control of the disclination lines is demonstrated by applying an electric field. The electric field rotates the 5CB director along the field and, thus, rearranges the disclination lines into an energetically more favourable state. As the particles are trapped by the disclination lines, their positions are also reconfigured. Figure 5a–d are POM images of the defect under the influence of a square-wave electric field (1 kHz) applied in the cell-normal direction. As the voltage is increased, spiralling wall disclinations (with the appearance of a thick thread) form in the surroundings and connect to the disclination lines, approximately halfway between the imprinted defect centres. As the elastic anisotropy of 5CB (ref. 30) causes regions with larger twists to have a larger Frederiks transition threshold^1^, a Frederiks transition front is initiated in the regions with the smallest twist and creates a twist wall at their boundaries (Supplementary Fig. 6). As seen in Fig. 5b–d, the walls change shape depending on the applied voltage and kinks appear in the disclination lines where the wall front is attached. Based on TPEM analysis, the wall front is located where the particles (and hence the disclination lines) are repelled from the bulk and attracted to either the top or the bottom substrates (Fig. 5e–h). Therefore, an electric field not only changes the particle positions two dimensionally, but also induces three-dimensional reconfiguration. Figure 5i–k shows the tuning range of the disclination network evaluated by measuring the two-dimensionally projected length of the disclination line and the vertical position of each particle at various applied voltages, where the cell gap is normalized to 1. Below the Frederiks transition threshold at 1.3 V, the two-dimensionally projected length of the disclination line remains almost unchanged (with variations smaller than 0.6%) and the particles within a single chain are displaced either to the top or bottom substrates. Above the threshold, the disclination network steeply shrinks, reducing its length by ∼12 % at 1.9 V (Fig. 5i). The vertical displacement behaviour also changes at the threshold and strongly depends on the position of the particle with respect to the wall front. The wall front position gradually shifts along the particle chain, and causes a reversal in the direction of vertical displacement; only the particles that have experienced a reversal in displacement direction show a steep change in position (Fig. 5j,k). For the two colloidal chains studied here, the third particle within the chain (particles 3 and 22) showed the largest tuning range, with the maximum shift reaching 41.1% of the cell thickness.

On further increasing the voltage, it is found that the walls undergo a ‘pincement' transition, in which a wall separates into two disclinations bound to each substrate confining the liquid crystal^31^ and extends outside the boundary of the alignment pattern (Supplementary Movie 3). The attraction of the particles to the top and bottom substrates observed in Fig. 5b–d is therefore considered to be a pre-transitional effect leading to the pincement transition. Above the pincement transition, the disclinations show a complex movement, which in some cases causes the particle positions to alternate within the disclination network, or be transported to outside the patterned region. However, the transient path of the disclinations depends on the manner in which the electric field is applied (for example, whether a voltage increase is applied instantaneously or gradually) and, although the disclination lines retain their original shapes when the voltage is removed, the particles often do not return to their original positions (Supplementary Movie 3). In contrast, below the pincement transition, the tuning of both disclination shapes and particle positions is fully reversible.

## Discussion

The giant electrical tunability of particle positions is a feature that has not been observed in other methods that allow control of particle positions in nematic liquid crystals. Patterned photoalignment (with non-topological patterns) and indented surfaces have been shown to induce localized elastic potentials at which a particle can be trapped^32^,^33^, introducing the possibility of controlling the crystallographic orientation of three-dimensional colloidal crystals^34^. However, localized elastic potentials induced by surface conditions are difficult to tune using an electric field, as the surface condition itself, which defines the elastic potential, remains unchanged. The use of bulk disclinations is a viable alternative for engineering the trapping potential landscape in liquid crystals, as they have large trapping potentials and can change shape easily in response to director deformations. Furthermore, the disclinations generated here are robust in that their existence is topologically protected and thus self-heal even from large distortions.

Our approach to engineering disclinations in nematic liquid crystals has a wide variety of applications, as the embedded particles are not limited to silica microspheres. For example, it would be possible to construct tunable chiral emitters or metamaterials by embedding fluorescent or metallic nanoparticles in the disclinations^16^,^35^,^36^. Such composites will not only possess the functions of the introduced particles but also show self-healing properties, as the disclination lines are topologically protected. Furthermore, the self-assembly behaviour of such colloidal composites can be tuned by changing the shape and topology of the introduced particles^9^,^37^. On the other hand, liquid crystalline materials may exhibit previously unseen mechanical, magnetic, or electronic properties as a result of containing ‘engineered' topological defects, as topology is known to affect such properties of a material^38^,^39^,^40^. Finally, another important avenue of investigation is the induction of artificial frustrated phases. Experiments and theory have shown that liquid crystals can sustain periodic or particle-like self-organized structures in the presence of defects^41^,^42^. The creation of disclination arrays with controlled numbers, lattice constants and symmetry may lead to the discovery of liquid crystal phases previously unknown.

## Methods

### Patterned photoalignment

Light from a high-pressure mercury lamp was filtered using a bandpass filter with centre wavelength of 436 nm and irradiated on a slit mask (Supplementary Fig. 1), which was imaged onto the sample using a pair of lenses with *f*=100 mm and *f*=18 mm. A motorized polarizer was placed between the two lenses and the sample stage was also motorized; the rotation speeds for the two stages were controlled as described in the main text using LabVIEW software. The sample was irradiated at an intensity of 0.3 mW for 60 s before being rotated by 3°, the apex angle of the slit mask.

### Fabrication of the liquid crystal cells

For the samples shown in Figs 1 and 2, the cells were fabricated from two 1-mm-thick glass substrates, onto which a layer of an azobenzene-based photoalignment material (DIC, LIA-03) was spin coated. This photoalignment layer provides ‘planar' liquid crystal alignment with the easy axis perpendicular to the polarization of the impinging light. The substrates were sandwiched temporarily using bead spacers of size 9 μm and patterned by photoalignment. The two substrates were offset after infiltrating the liquid crystal 5CB, which shows the nematic liquid crystal between 22 °C and 35 °C. The observed colour is determined by the optical retardation of the sample, which is affected by the actual cell gap and observation temperature. The samples were observed at different temperatures to illustrate the stability of the alignment as well as to emphasize the difference in the texture as follows: Fig. 1a–d, 34 °C, 29 °C, 30 °C and 32 °C, respectively, and Fig. 2a–c, 34 °C, 33 °C and 30 °C, respectively.

For the samples shown in Figs 3, 4, 5, a 150-μm-thick cover slip was used as one of the substrates for the purpose of performing TPEM. Substrates coated with a 100-nm-thick layer of indium tin oxide were used for the sample shown in Fig. 5, to apply a voltage. The samples for Figs 3 and 5 were fabricated by offsetting the defect patterns after a single photoalignment process, whereas the sample for Fig. 4 was fabricated by patterning the two substrates independently and sandwiching them afterwards to a lateral separation distance of 100 μm. The liquid crystal was doped with silica particles (JGC Catalysts and Chemicals, Shinshikyu SW 3 μm) and a dichroic dye (Exciton, DCM), both at a concentration of 1 wt%. The particles were coated with DMOAP following the procedure described in ref. 28.

### Two-photon excitation microscopy

TPEM is a fluorescence imaging technique which uses two-photon absorption to achieve three-dimensional resolution^25^. The contrast in fluorescence intensity between the dye-doped liquid crystal and the non-fluorescent silica particles allows detection of the particle distribution within the specimen. A commercial confocal laser scanning microscope (Zeiss, LSM-510) was used in conjunction with a titanium-sapphire laser (Spectra Physics, Maitai) with wavelength of 800 nm, pulse width of 150 fs and repetition rate of 82 MHz. An oil-immersion objective lens with magnification of × 63 and numerical aperture of 1.4 was used to acquire the images.

### Simulation of the defect profile

The defect profiles were calculated by minimizing the free energy of the liquid crystal cell as a functional of the orientational order parameter of second-rank tensor (*Q*_*ij*_). The free energy after appropriate rescaling is expressed as 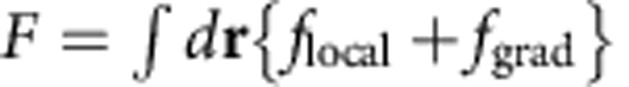, where *f*_local_=(1/2)*A*Tr*Q*^2^–(1/3)*B*Tr*Q*^3^+(1/4)*C*(Tr*Q*^2^)^2^, with 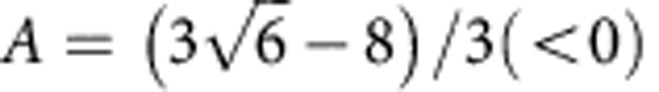, 
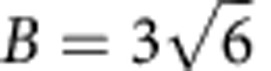
 and *C*=4, which is the local part of the free energy density (Tr is the trace of a tensor), and *f*_grad_=(1/2)*L*_1_(∇ × *Q*)_*ij*_(∇ × *Q*)_*ij*_+(1/2)*L*_2_(∇·*Q*)_*j*_(∇·*Q*)_*j*_, which is the elastic free-energy density due to the spatial inhomogeneity of *Q*_*ij*_. Here, summations over repeated indices are implied, (∇ × *Q*)_*ij*_≡*ɛ*_*ist*_∇_*s*_*Q*_*tj*_ and (∇·*Q*)_*j*_≡∇_*i*_·*Q*_*ij*_, where *ɛ*_*ist*_ is the Levi–Civita antisymmetric symbol. It is noteworthy that setting *L*_1_=*L*_2_ corresponds to the so-called one-constant approximation (*K*_11_=*K*_22_=*K*_33_, where *K*_11_, *K*_22_ and *K*_33_ are the splay, twist and bend elastic constants, respectively^6^). Here we set *L*_1_=0.2 and *L*_2_=0.8, corresponding to *K*_22_/*K*_11_=*K*_22_/*K*_33_=0.4. It is also worth noting that *Q*_*ij*_=*Q*_0_(*n*_*i*_*n*_*j*_–(1/3)*δ*_*ij*_) with *Q*_0_=1 minimizes *F*. See refs 42, 29 for the free energy and its rescaling. The length has been rescaled so that the rescaled unit length corresponds to nanometres, where nanometres is the nematic coherence length^29^.

The two confining surfaces (parallel to the *xy* plane) impose Dirichlet boundary conditions fixing *Q*_*ij*_ there, corresponding to the strong anchoring conditions in the experiments. We set *Q*_*ij*_=*Q*_0_(*n*_*i*_*n*_*j*_–(1/3)*δ*_*ij*_), where *Q*_0_=1 and **n**=(cos Θ(*x*, *y*), 0). The angle Θ(*x*, *y*) is chosen so that it reproduces the experimental surface orientation profiles described in the main text. The other boundaries (parallel to the *xz* or *yz* plane) impose no surface energy.

The free-energy functional is minimized on a cubic lattice of dimension 640 × 640 × 20, with the lattice spacing equal to 1 in the rescaled unit. Offset of the two defects is set to 100, 5 times as large as the cell thickness. We let the initial profile *Q*_*ij*_=0 (except at the confining surfaces) relax via a simple rotational relaxation equation, *∂Q*_*ij*_(**r**)/*∂t*=−*δF/δQ*_*ij*_(**r**)+*λδ*_*ij*_, where the Lagrange multiplier *λ* ensures Tr*Q*=0. In the figures, defects are defined by the regions with weaker orientational order and are identified by the isosurfaces Tr*Q*^2^=0.6. Although the thickness of the simulation box, ≅0.8 μm, is much smaller than that of the experimental cell, the former can be further rescaled to fit the latter as long as the director profile **n** and the position of the resulting disclinations are concerned. It is because in the bulk of the nematic liquid crystal with uniaxial order, the elastic energy in terms of the tensor-order parameter *Q*_*ij*_ reduces to the Frank elastic energy in terms of **n** that does not possess any characteristic lengths. Furthermore, rigid surface anchoring (fixed *Q*_*ij*_) in our simulations does not introduce any additional lengths either (anchoring extrapolation length is zero). This is the reason why our simulations successfully reproduce the shape of the disclinations observed experimentally.

## Additional information

**How to cite this article:** Yoshida, H. *et al*. Three-dimensional positioning and control of colloidal objects utilizing engineered liquid crystalline defect networks. *Nat. Commun.* 6:7180 doi: 10.1038/ncomms8180 (2015).

## Supplementary Material

Supplementary Information Supplementary Figures 1-6 and Supplementary References

Supplementary Movie 1 Disclination generation by offsetting of topogical patterns. Two substrates patterned using a linear bow-tie slit having (s, c) = (1, 0) are gradually separated. As soon as the two defect centers are separated, two disclination lines connect the two defect centers, and the pattern increases in size as the separation of the defect centers increases. The movie has been accelerated by a factor of 2.

Supplementary Movie 2 Observation of three-dimensional particle distribution. The intrinsic three-dimensional resolving capability of two-photon excitation microscopy allows images to be acquired at different focal planes within the sample. As the focus is gradually scanned through the sample, the arrangement of the particles in focus changes, indicating that the arrangement is three-dimensional. The fluorescence intensity profile measured along the disclination path corresponds to Fig. 3c in main text.

Supplementary Movie 3 Pincement transition causing complex disclination movement. An electric field is applied between the two substrates confining the liquid crystal layer patterned with spiral defects of (s, c) = (1, 0). The applied voltage (a square wave with frequency 1 kHz) is scanned gradually from 0 V to 5 V and back. The wall undergoes a pincement transition and separates into two disclination lines that extend outside the boundary of the alignment pattern. The disclinations show a complex movement such that the disclinations cross each other and become elongated in the vertical direction (in contrast to the initial state in which the disclination spans horizontally). The deformation is reversible and returns to its initial state when the voltage is removed. However, the particles that existed on the lower half disclination line is rotated to the upper half disclination through this process, indicating that the particle positions can be alternated within the disclination network. The movie has been accelerated by a factor of 3.
