# Supplementary material for: Three-dimensional positioning and control of colloidal objects utilizing engineered liquid crystalline defect networks
Source: Nat Commun. 2015 May 21;6:7180. doi: 10.1038/ncomms8180 (PMC4647884; doi:10.1038/ncomms8180)
Supplement: Supplementary Information — Supplementary Figures 1-6 and Supplementary References [file ncomms8180-s1.pdf]

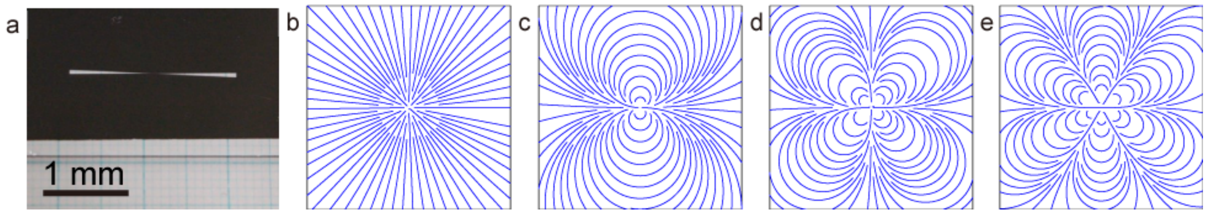

Supplementary Figure 1: Orientational patterns created from a linear slit. a Photograph of the straight bow-tie-shaped slit. The slit is 2mm long, with an opening of 3 °. **b-e**, Streamline plots of the orientational patterns created by using the straight bow-tie-shaped slit, for topological charges or defect strengths of 1 (**b**), 2 (**c**), 3 (**d**), and 4 (**e**). The patterns were calculated using the expression described in the main text, and the nematic liquid crystal aligns along the streamlines.

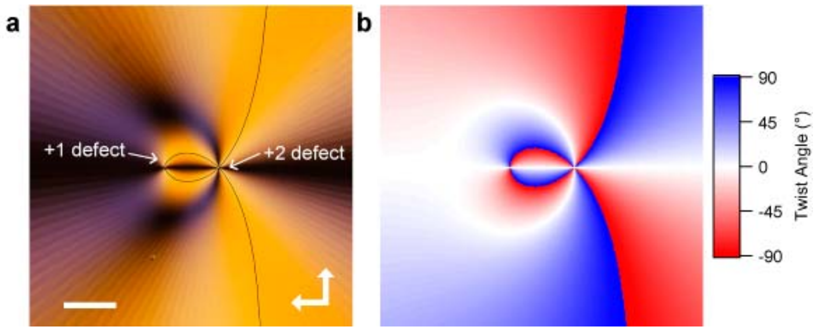

Supplementary Figure 2: Disclinations in a system with unequal defect strengths. a, POM image of sample with (s, c) = (1, 0) and (s, c) = (2, 0). The s = 2 defect produces 4 disclinations, whereas the s = 1 defect produces only two. The total number of disclinations is determined by the defect with the larger strength. Scale bar: 100 µm. **b** Calculation of the twist angle, showing excellent agreement with the obtained disclination pattern.

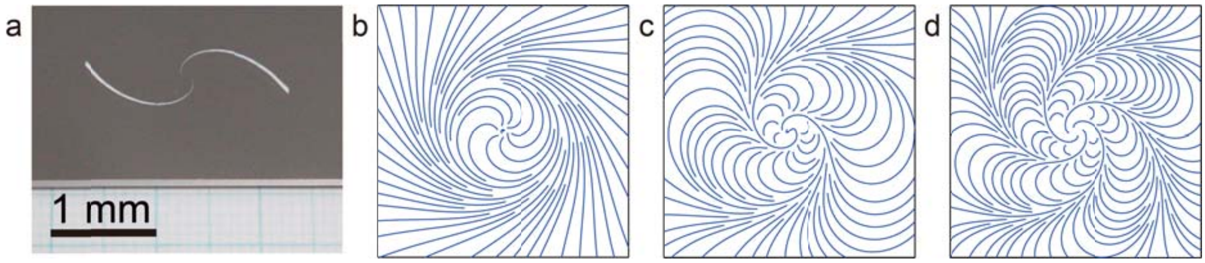

Supplementary Figure 3: Orientational patterns created from a spiral slit. **a** Photograph of the spiral bow-tie-shaped slit. The slit is 2mm long, with an opening of 3 °. **b-d**, Streamline plots of the orientational patterns created by using the spiral bow-tie-shaped slit, for topological charges or defect strengths of 1 (**b**), 2 (**c**), amd 3 (**d**). The patterns were calculated using the expression described in the main text, and the nematic liquid crystal aligns along the streamlines.

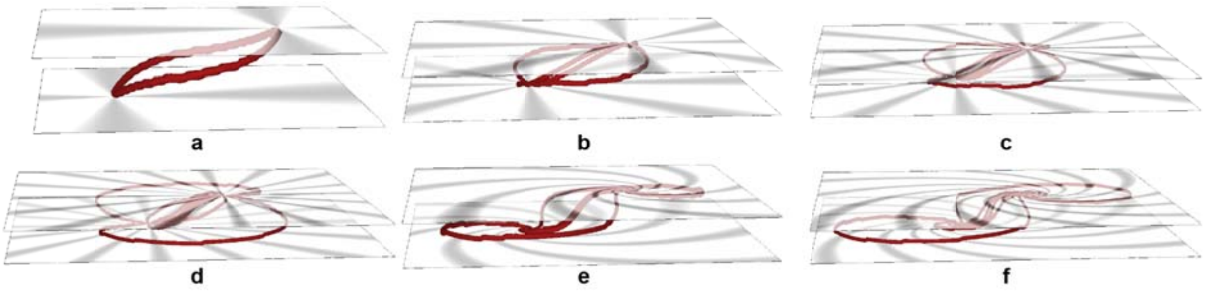

Supplementary Figure 4: Landau-de Gennes free energy modelling of disclination shapes. a-d, Disclination network generated between two substrates patterned with a linear bow-tie-shaped slit with the same (s, c) values. (s, c) = (1, 0) (**a**), (s, c) = (2, 0) (**b**), (s, c) = (3, 0) (**c**), and (s, c) = (4, 0) (**d**). The calculation conditions are the same as that described in the Methods Section. **e,f,** Disclination network generated between two spirally patterned substrates with the same (s, c) values. (s, c) = (2, 0) (**e**), and (s, c) = (3, 0) (**f**). Note that calculations were performed assuming a continuous distribution of surface anchoring, in contrast to the segmented pattern (due to the 3° rotation step of the motorized stages in the photoaligning setup) that was actually created in experiment. We have confirmed that the 3° segmentation has negligible effect on the shape of the disclination, as the disclinations mostly exists in the bulk where the director field becomes continuous and the discontinuity arising from the segmented surface alignment is smeared out.

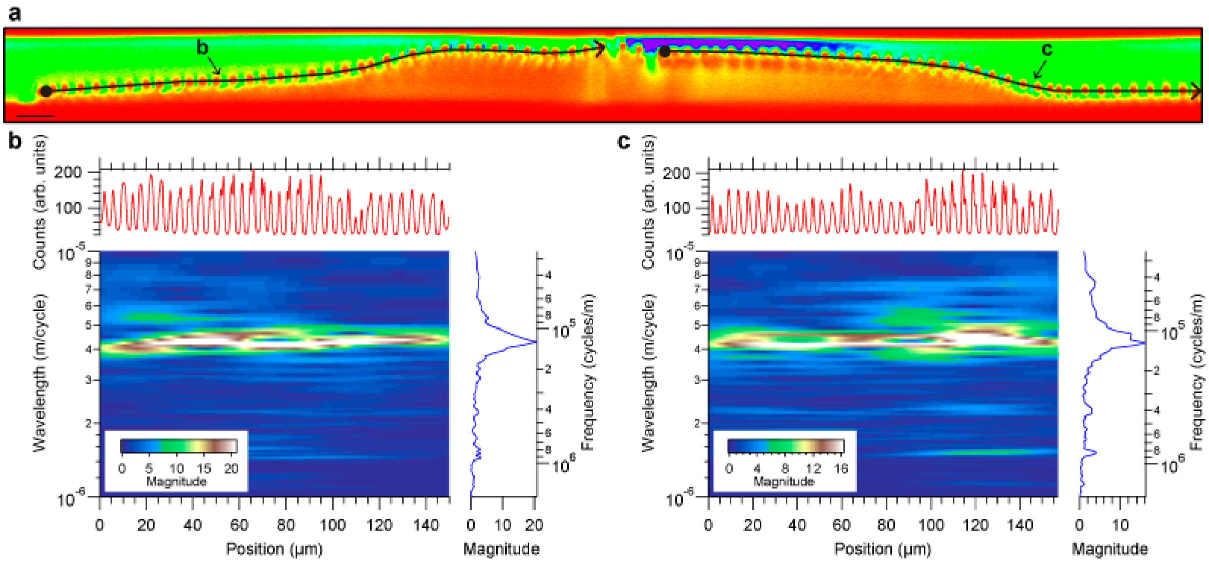

Supplementary Figure 5: Space-frequency analysis of the colloidal particle chain. a, Cross-sectional TPEM image of colloidal superstructure built using spirally patterned substrates with (*s*, *c*) = (1, 0). The figure is identical with Fig. 3c except that the path along which the space-frequency analyses were performed is overlaid. **b,** Space-frequency analysis of the colloidal particle chain. Calculations were performed using the Time-frequency Toolkit for Igor Pro software. *Top*: TPEM profile along particle chain marked by 'b' in Supplementary Fig. 4a. *Center*: Gaussian Wigner Transform calculated from the particle profile. The width of the Gaussian to smooth the decomposition is set to half of the full data width in the time domain, and is set to fulfill the minimum uncertainty condition in the frequency domain. *Right*: Spectral estimate calculated by taking the mean of the Gaussian Wigner Transform. **c,** Space-frequency analysis of the colloidal particle chain along marked by 'c' in Supplementary Fig. 4a. For both chains, the inter-particle spacing is approximately constant along the chain.

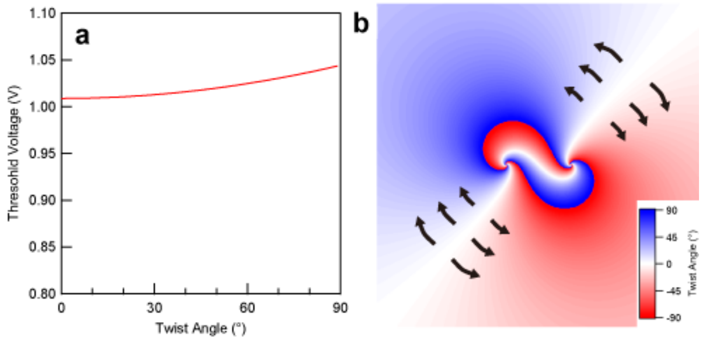

Supplementary Figure 6: Frederiks transition in a twisted nematic liquid crystal. a, Dependence of the Frederiks transition threshold V_c on the twist angle ϕ, calculated according to the equation $V_c=\pi((K_{11} + (K_{33}-2K_{22})(\phi/\pi)^2)/\varepsilon_0\varepsilon_a)^{1/2}$, where K_{11}, K_{22} and K_{33} are splay, twist and bend constants, respectively, and ε_a is the dielectric anisotropy[1]. Material parameters were taken from literature[2,3] and are as follows: K_{11} = 10.5 pN, K_{22} = 5.4 pN, K_{33} = 13.8 pN, $\Delta\varepsilon$ = 11.5. **b,** The difference in the Frederiks transition threshold causes a 'front' to initiate from regions with the smallest twist angle. A twist wall results where the fronts meet.

Supplementary References:

1. Yeh, P. & Gu, C. *Optics of liquid crystal displays*. (Wiley, 2010).
2. Madhusudana, N. V. & Pratibha, R. Elasticity and orientational order in some cyanobiphenyls: Part IV. Reanalysis of the data. *Mol. Cryst. Liq. Cryst.* **89,** 249–257 (1982).
3. Belyaev, B. A., Drokin, N. A., Shabanov, V. F. & Shepov, V. N. Dielectric anisotropy of 5CB liquid crystal in a decimeter wavelength range. *Phys. Solid State* **42,** 577–579 (2000).
